# Causal association between 637 human metabolites and ovarian cancer: a mendelian randomization study

**DOI:** 10.1186/s12864-024-09997-3

**Published:** 2024-01-23

**Authors:** Yedong Huang, Wenyu Lin, Xiangqin Zheng

**Affiliations:** 1 College of Clinical Medicine for Obstetrics & Gynecology and Pediatrics, Fujian Medical University, Fuzhou, China; 2 Department of Gynecology Oncology, Fujian Maternity and Child Health Hospital, Affiliated Hospital of Fujian Medical University, Fuzhou, China; 3 Laboratory of Gynecologic Oncology, Fujian Maternity and Child Health Hospital, Affiliated Hospital of Fujian Medical University, Fuzhou, China; 4 Fujian Province Key Clinical Specialty for Gynecology, Fujian Key Laboratory of Women and Children’s Critical Disease Research, National Key Gynecology Clinical Specialty Construction Insititution of China, Fuzhou, China

**Keywords:** Mendelian randomization, Ovarian cancer, Metabolites, Casual effect

## Abstract

**Background:**

Current evidence suggests a significant association between metabolites and ovarian cancer (OC); however, the causal relationship between the two remains unclear. This study employs Mendelian randomization (MR) to investigate the causal effects between different metabolites and OC.

**Methods:**

In this study, a total of 637 metabolites were selected as the exposure variables from the Genome-wide Association Study (GWAS) database (http://gwas.mrcieu.ac.uk/datasets/). The OC related GWAS dataset (ieu-b-4963) was chosen as the outcome variable. R software and the TwoSampleMR package were utilized for the analysis in this study. MR analysis employed the inverse variance-weighted method (IVW), MR-Egger and weighted median (WM) for regression fitting, taking into consideration potential biases caused by linkage disequilibrium and weak instrument variables. Metabolites that did not pass the tests for heterogeneity and horizontal pleiotropy were considered to have no significant causal effect on the outcome. Steiger’s upstream test was used to determine the causal direction between the exposure and outcome variables.

**Results:**

The results from IVW analysis revealed that a total of 31 human metabolites showed a significant causal effect on OC (*P* < 0.05). Among them, 9 metabolites exhibited consistent and stable causal effects, which were confirmed by Steiger’s upstream test (*P* < 0.05). Among these 9 metabolites, *Androsterone sulfate*, *Propionylcarnitine*, *5alpha-androstan-3beta,17beta-diol disulfate*, *Total lipids in medium VLDL* and *Concentration of medium VLDL particles* demonstrated a significant positive causal effect on OC, indicating that these metabolites promote the occurrence of OC. On the other hand, *X-12,093*, *Octanoylcarnitine*, *N2,N2-dimethylguanosine*, and *Cis-4-decenoyl carnitine* showed a significant negative causal association with OC, suggesting that these metabolites can inhibit the occurrence of OC.

**Conclusions:**

The study revealed the complex effect of metabolites on OC through Mendelian randomization. As promising biomarkers, these metabolites are worthy of further clinical validation.

**Supplementary Information:**

The online version contains supplementary material available at 10.1186/s12864-024-09997-3.

## Background

Ovarian cancer (OC) is the sixth most common cancer in women of developed countries [1]. It is important to note that OC is the most fatal of all gynecologic cancers and accounts for more than two thirds of all deaths due to gynecologic cancers [2], with a 5-year relative survival rate of 49% [3]. This is partly due to the fact that 70% of OC patients are diagnosed in advanced stage when they receive treatment for the first time, as there are no specific symptoms of OC in primary stages [4]. At present, early diagnosis of OC is dependent on B-ultrasound and serum cancer antigen 125 (CA125) detection. However, sensitivity of CA125 drops significantly in early-stage disease and in premenopausal women [5]. One of the most fundamental differences between cancer and non-malignant cells is their metabolism [6]. A large number of cancer-promoting components such as metabolites in ascites were reported to promote OC invasion and resistance to chemotherapy through surface-specific receptors on tumor cells [7]. Therefore, numerous studies have aimed to improve diagnosis and treatment of OC using metabolomics.

Metabolomics, which is the metabolites profiling in biological matrices, is a key tool for biomarker discovery and personalized medicine and has great potential to elucidate the ultimate product of the genomic processes [8]. Recently, it has been successfully utilized to find new biomarkers and investigate pathogenesis in OC. Metabolomics analysis revealed association of some biomarkers, including *lysophosphatide* and *adrenaline glycosamide* with epithelial ovarian cancer (EOC) [9]. Chen et al. identified and validated 2*7-nor-5β-cholestane-3,7,12,24,25 pentol glucuronide* as a diagnostic marker that is complementary to CA125 [10]. *Sphingomyelins* 3–23 years before diagnosis were associated with increased risk of OC, regardless of histotype, with stronger associations among postmenopausal women [11]. To date, metabolic profiling for OC has been performed with relatively small sample sizes that limit the robustness and statistical significance of the results. In addition, most studies have had relatively limited follow-up with participants, which may be insufficient for detecting risk factors on cancer outcomes that take more than 10 years to develop.

An approach to overcome the potential limitations of observational epidemiology and strengthen the evidence for a potential causal role of metabolite on OC risk is MR. In a MR study, genetic variants associated with an exposure of interest are identified from GWAS and subsequently applied to an independent data set to derive an unbiased estimate of the exposure-outcome association [12]. The MR study design offers several advantages over traditional epidemiological studies. First, MR can avoid the bias caused by reverse causality to a certain extent [13]. Second, MR studies are independent to common behavioral, physiological, and socioeconomic confounders owing to random assignment of alleles at meiosis. Third, MR design resembling randomized controlled trial (RCT) significantly reduced concerns in terms of ethical, applicability, and financial issues [14].

In this study, we employed MR analysis to conduct a summary statistical analysis of the GWAS data in European populations, aiming to determine the causal effects of 637 human metabolites on OC.

## Materials and methods

### Data source and software preparation

The dataset corresponding to human metabolites is derived from the GWAS database (http://gwas.mrcieu.ac.uk/datasets/), which includes a total of 637 metabolites. The dataset associated with OC is encoded as ieu-b-4963 in the GWAS database. The MR analysis in this study primarily relied on the TwoSampleMR package in R software (version 4.1.3). Moreover, for the MR analysis and the graphical representation of the outcomes, a suite of R packages were utilized, encompassing “ieugwasr, plinkbinr, ComplexHeatmap, circlize, dendextend, dendsort, tidyverse, and ggforestplot”.

### Instrument variable selection

The selection criteria for single nucleotide polymorphisms (SNPs) associated with the 637 metabolites are based on the following criterion: *P* < 5*10^− 6^. To ensure the independence among SNPs, i.e., adherence to the Mendelian second law, this study set the parameters for linkage disequilibrium as follows: r^2^ < 0.001 and kb > 10,000. SNPs selected based on these criteria are considered instrumental variables (IVs) and are included in subsequent analyses.

### Exclusion of weak IVs

To ensure the accuracy of the results, this research employs the F-statistic as a measure to reflect the degree of association between instrumental variables and the exposure. The formula for calculating the F-statistic is as follows: F = (β/SE) ^2^. In this study, an evaluation criterion for weak IVs is set as F < 10. Given the first assumption of MR analysis, which is the assumption of instrument relevance, it is necessary to ensure a significant association between IVs and the exposure. Therefore, weak IVs selected based on the aforementioned method should be excluded.

### Data analysis

The analysis in this study was conducted using the TwoSampleMR package in R software (version 4.1.3). The IVW results was utilized to determine whether there is a significant causal effect between the exposure and the outcome. Building upon this, exposures identified as positive (*P* < 0.05) were further subjected to False Discovery Rate (FDR) testing to minimize the risk of Type I errors, or false positives. The FDR analysis was also conducted using the R software (version 4.1.3). Concurrently, with the aim of assessing the frequency of Type II errors, this study made use of the mRnd online resource (https://shiny.cnsgenomics.com/mRnd/) to perform calculations of statistical power [15]. During the calculation of statistical power, the parameter for the Type I error rate (α) was set to 0.05.

To mitigate potential biases introduced by individual models, the weighted median and MR-Egger methods were employed as supplementary and reference approaches to the IVW model. If the causal effect directions are consistent across these three models, it suggests a relatively stable causal effect between the exposure and the outcome. *P* < 0.05 was considered statistically significant.

### Heterogeneity, horizontal pleiotropy, Steiger upstream test and PhenoScanner

The Cochran Q test and MR-Egger intercept test were employed in this study to assess heterogeneity and horizontal pleiotropy, respectively. Heterogeneity represents the variability in the causal effect of SNPs associated with the exposure on the outcome. If heterogeneity is significant, it suggests an unstable causal effect. Horizontal pleiotropy indicates the possibility that SNPs directly affect the outcome through factors other than the exposure. If horizontal pleiotropy is significant, it implies that the causal effect is not valid. The Steiger upstream test, also known as the Steiger filtering method, is used to assess the directionality of the exposure on the outcome. If the Steiger test yields a significant result, it indicates that the exposure is upstream of the outcome, confirming the correct directionality. In all three test methods mentioned, a significance level of *P* < 0.05 is considered statistically significant. Additionally, the results obtained from the PhenoScanner database [16] (http://www.phenoscanner.medschl.cam.ac.uk/) serve as an important reference for ruling out horizontal pleiotropy of this study.

## Results

### Data and detailed information

**Fig. 1:**
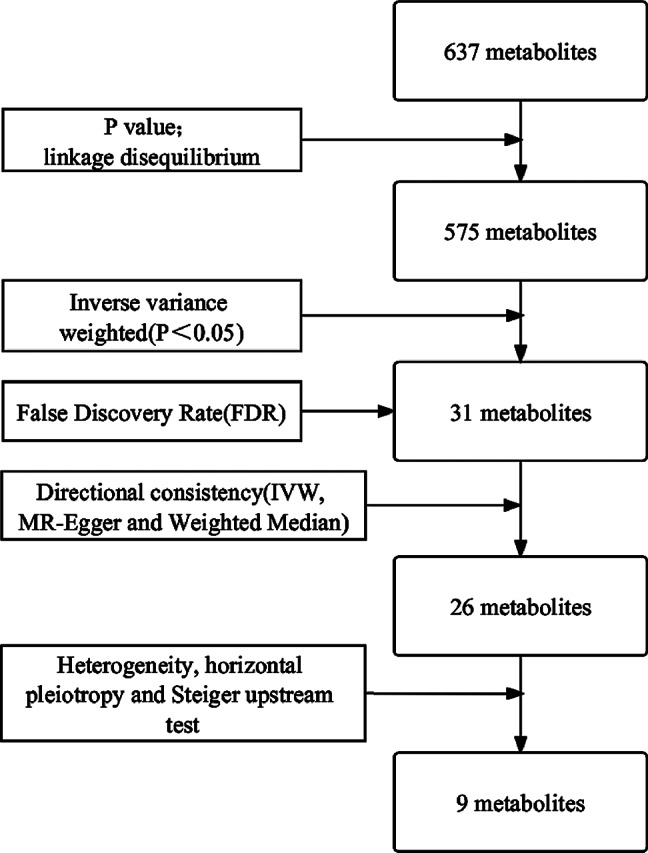
Flow chart for filtering metabolites

The metabolite-related GWAS dataset used in this study is primarily based on the genetic locus study conducted by Shin et al. [17] in 2014, which investigated the impact of genetic variations on human metabolism, as well as the genome-wide study on human circulating metabolites conducted by Kettunen et al. [18] in 2016. The dataset comprising 637 metabolites was presented in detail in Supplementary Material 1 (Suppl 1. Detailed Information of 637 Human Metabolite Data). The GWAS dataset corresponding to OC, encoded as ieu-b-4963, was derived from the sequencing results of 199,741 samples conducted by Burrows et al. in 2021. All the metabolite datasets and the OC related dataset were based on sequencing performed on individuals of European ancestry. Figure 1 illustrates the workflow for metabolite selection in this study.

### IVs selection and exclusion of weak IVs

The GWAS dataset for all metabolites was screened for SNPs based on the criterion of *P* < 5*10^− 6^. The parameters for linkage disequilibrium were set as r^2^ < 0.001 and kb > 10,000. If a metabolite had an insufficient number of SNPs for MR analysis, it was excluded from the study. The SNPs selected based on the criterion of F statistic ≥ 10 are presented in detail in Supplementary Material 2 (Suppl 2. List of Filtered SNPs). The F statistics for all SNPs ranged from 19.1702 to 508.7844.

### MR analysis and test results

**Fig. 2:**
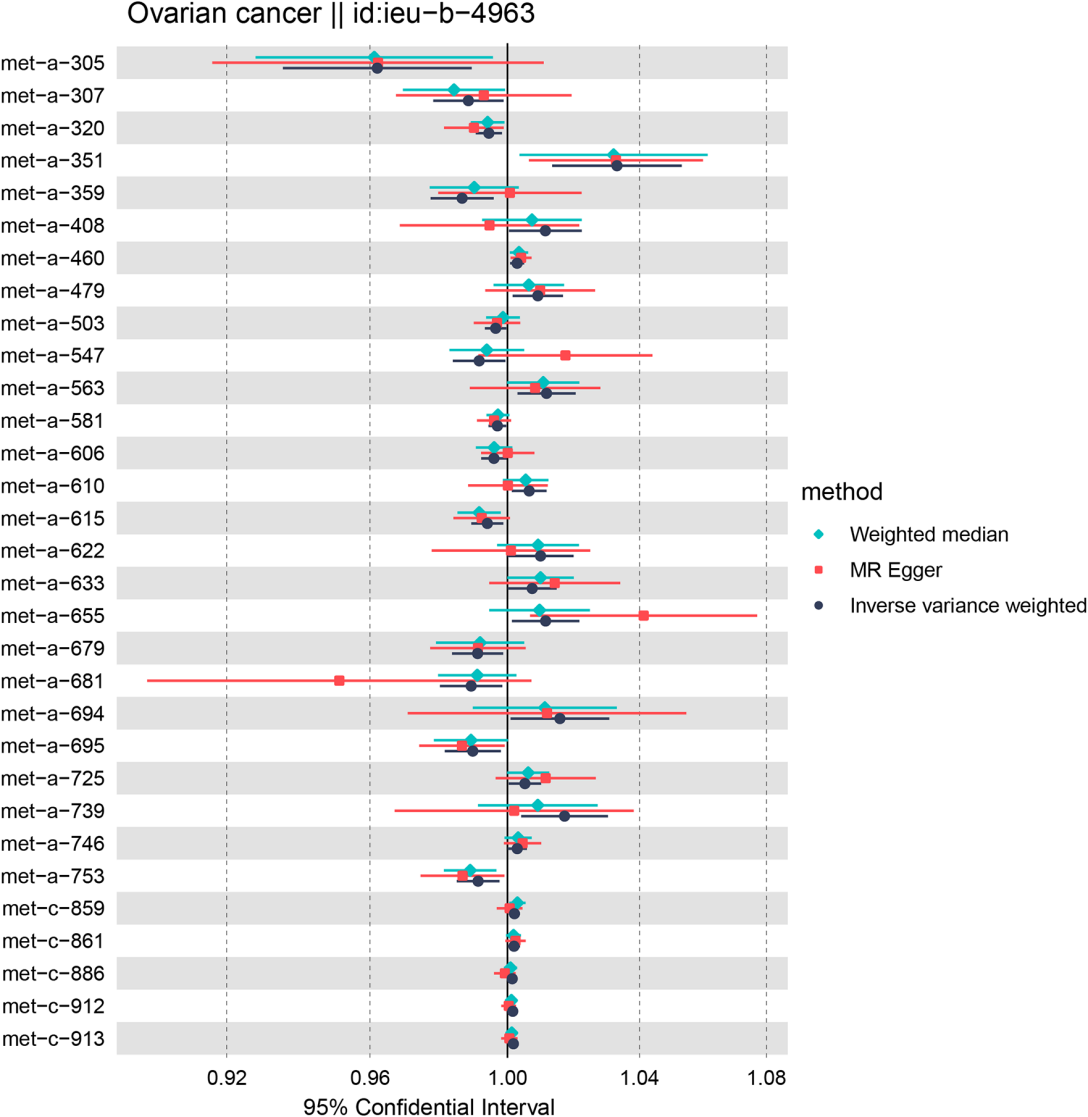
Forest plot for MR results of 31 metabolites

As shown in Fig. 2, the MR analysis revealed a potential causal effect between 31 metabolites and OC (P-value of IVW < 0.05). Following FDR analysis, no exposures were excluded due to adjusted P-values ≥ 0.05. The analysis process of the FDR-adjusted P-values is displayed in Supplementary Material 3 (Suppl 3. FDR-adjusted P-values of 31 Metabolites). Supplementary Material 4 (Suppl 4. IVW Analysis Results and Statistical Power Values for 31 Metabolites) presents the results of the statistical power calculations. It is noteworthy that the statistical power for most metabolites is not optimal, indicating a higher probability of committing Type II errors. In the context of this study, we prioritize a lower probability of Type I errors, which inevitably compromises some degree of statistical power. Faced with the trade-off between Type I and Type II error rates, our focus is predominantly on minimizing the occurrence of Type I errors.

**Fig. 3:**
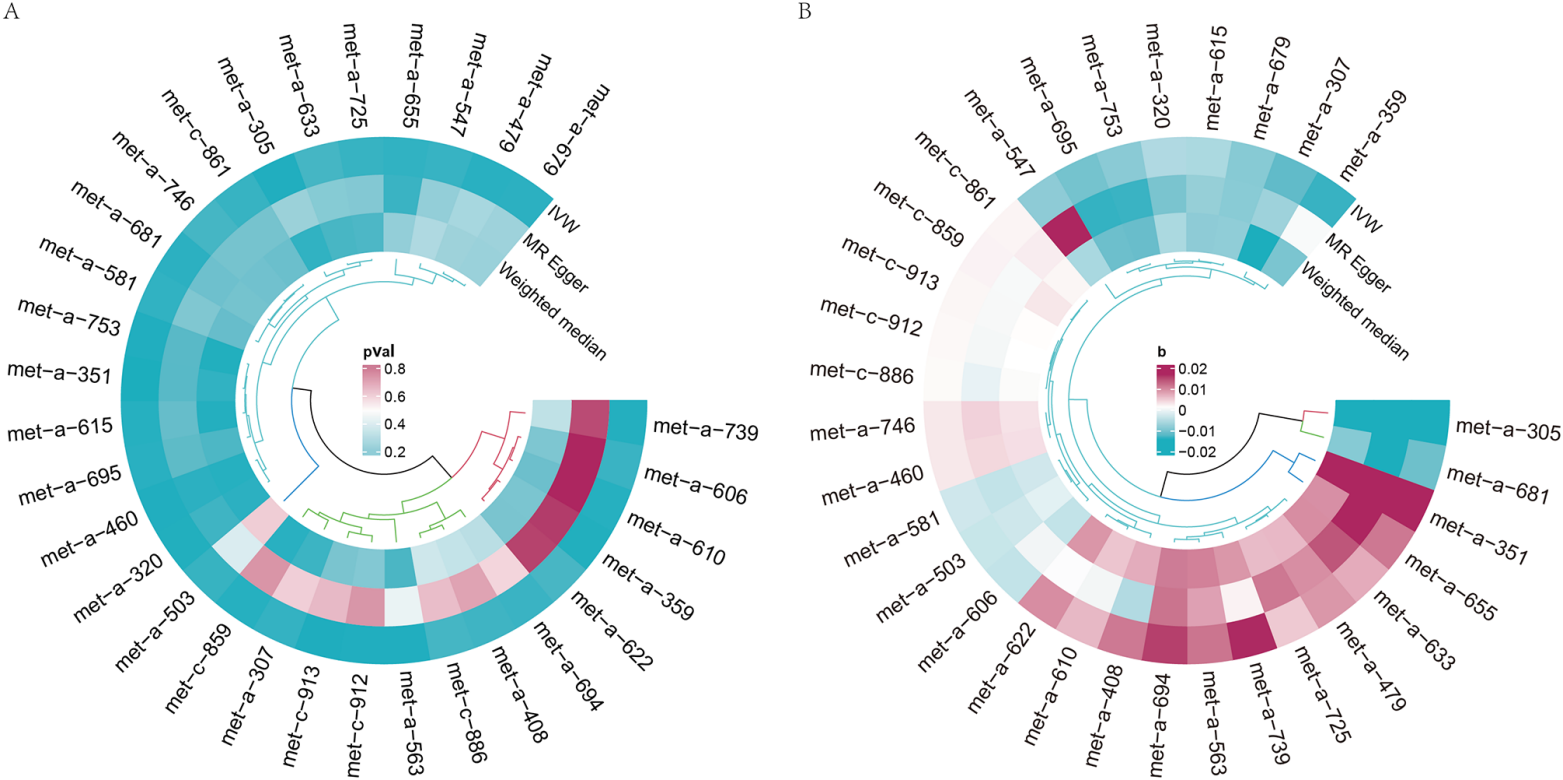
The circular heatmap shows the P values and β values of algorithms corresponding to 31 metabolites. (**A**) Heatmap of P values corresponding to 31 metabolites. (**B**) Heatmap of β values corresponding to 31 metabolites *Note*:The clustering algorithm used in the circular heatmap is an unsupervised clustering method, which simply represents the similarity among the data points

Figure 3 presents a circular heatmap illustrating the P values and β values of IVW, MR-Egger and weighted median methods corresponding to the aforementioned metabolites (Fig. 3A and B). Among them, 26 metabolites showed consistent causal effects with the IVW direction based on the weighted median and MR-Egger. Furthermore, 9 metabolites successfully passed tests for heterogeneity, horizontal pleiotropy, and exhibited the correct causal effect direction in the Steiger test. Table 1 displays the 9 metabolites that passed the tests along with their respective P-values. Supplementary Material 5 (Suppl 5. Results of PhenoScanner) presents the results retrieved from the PhenoScanner database, which do not show significant horizontal pleiotropy. It is noteworthy that currently, there is no method that can completely eliminate the influence of horizontal pleiotropy, and the interpretation of the results from PhenoScanner also carries a certain degree of subjectivity.

**Table 1:** Details of 9 metabolites that passed the test

ID	P value of heterogeneity(IVW)	P value of heterogeneity(MR-Egger)	P value of horizontal pleiotropy	P value of Steiger test	Direction
met-a-460	0.8771803	0.8869046	0.3295969	1.00E-07	Forward
met-a-479	0.9631611	0.9478844	0.9274689	0.0232969	Forward
met-a-581	0.4351166	0.367689	0.6573945	1.20E-06	Negative
met-a-615	0.9353139	0.9141247	0.643113	2.57E-05	Negative
met-a-679	0.9930601	0.9899221	0.9868937	0.0463142	Negative
met-a-746	0.5434027	0.5008567	0.519298	0.0442872	Forward
met-a-753	0.7539987	0.7425412	0.4255294	0.0009622	Negative
met-c-912	0.8923032	0.9145709	0.2126228	0.0196859	Forward
met-c-913	0.728432	0.7458959	0.2694708	0.0208481	Forward

### Scatter plots of MR analysis

**Fig. 4:**
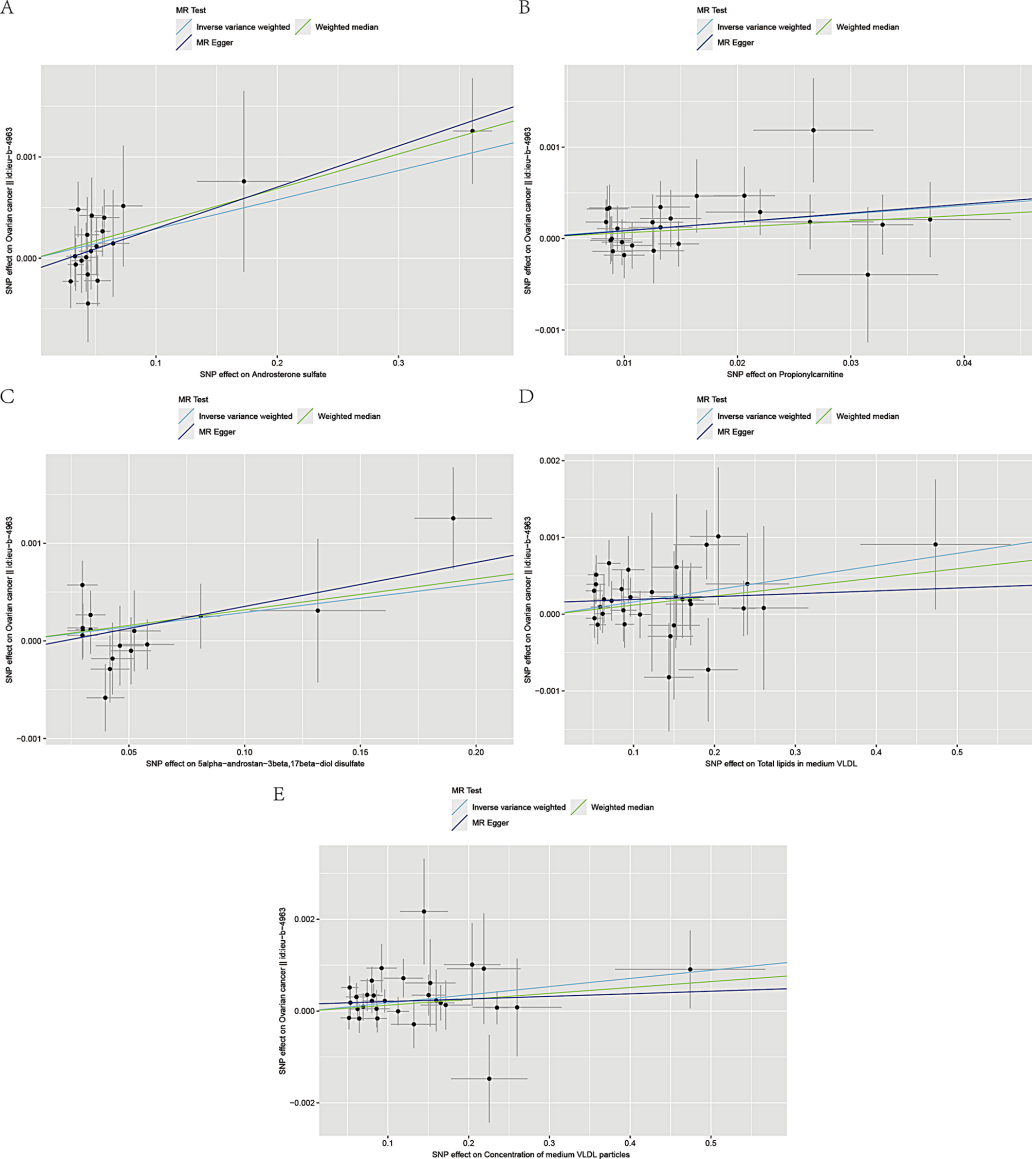
Scatter plots of 5 metabolites with forward direction

**Fig. 5:**
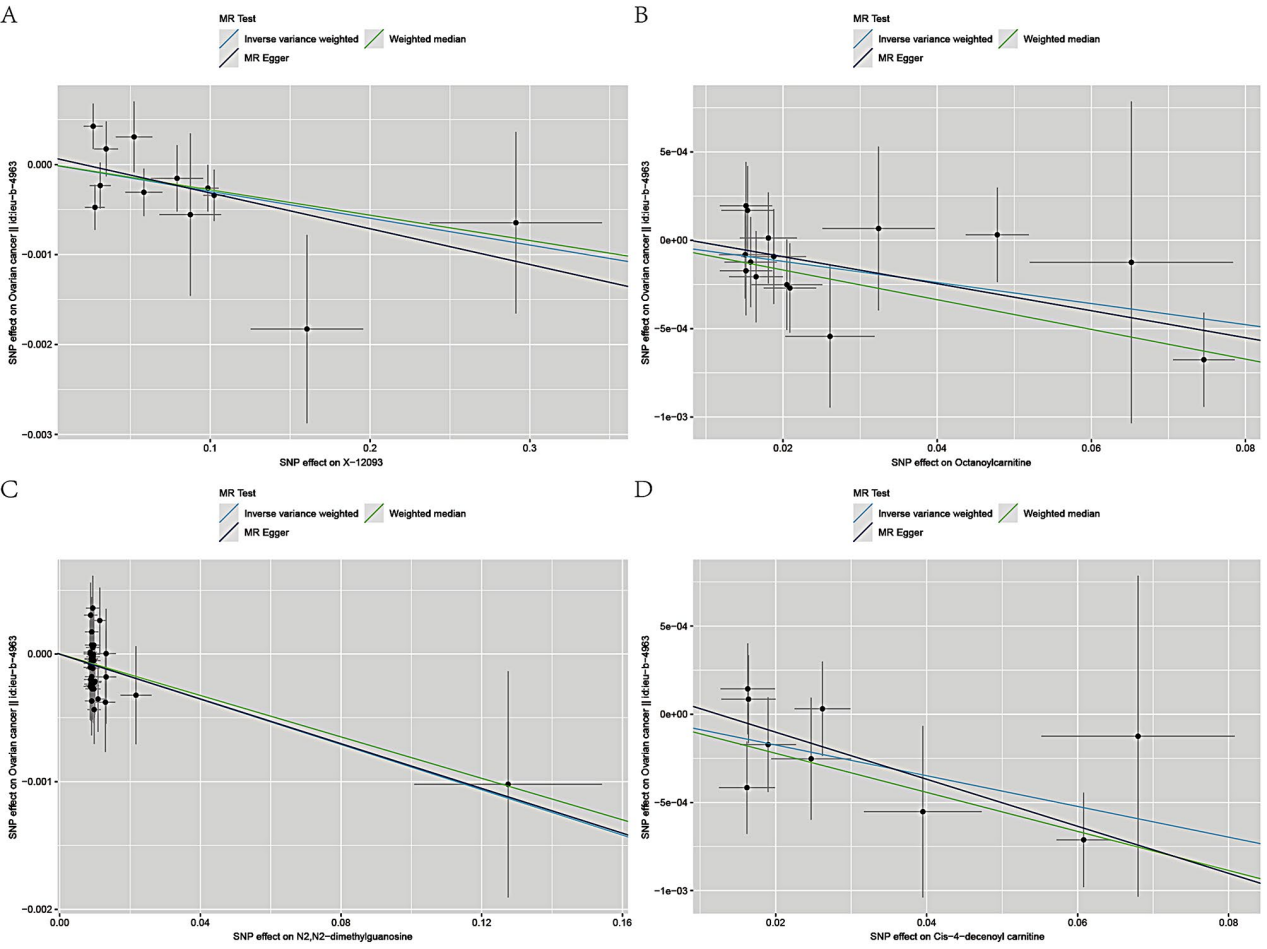
Scatter plots of 4 metabolites with negative direction

Figures 4 and 5 present the regression results of the SNPs corresponding to the aforementioned 9 metabolites using the IVW, weighted median and MR-Egger algorithms. Figure 4 indicates that *androsterone sulfate* (Fig. 4A), *propionylcarnitine* (Fig. 4B), *5alpha-androstan-3beta,17beta-diol disulfate* (Fig. 4C), *total lipids in medium VLDL* (Fig. 4D), and *concentration of medium VLDL particles* (Fig. 4E) exhibit significant positive causal effects on OC. Conversely, *X-12,093* (Fig. 5A), *octanoylcarnitine* (Fig. 5B), *N2,N2-dimethylguanosine* (Fig. 5C), and *cis-4-decenoyl carnitine* (Fig. 5D) show significant negative causal associations with OC.

## Discussion

OC is a silent cancer which rate survival mainly relays in early stage detection. The discovery of reliable OC biomarkers plays a crucial role in the disease management and strongly impact in patient’s prognosis and survival. In this study, we used MR method to determine the casual relationship between 637 metabolites and OC. Specifically, *androsterone sulfate*, *propionylcarnitine*, *5alpha-androstan-3beta-17beta-diol disulfate*, and *medium VLDL* may promotes OC oncogenesis. While *X-12,093*, *octanoylcarnitine*, *N2,N2-dimethylguanosine*, and *cis-4-decenoyl carnitine* may suppress OC.

Accumulating evidence has demonstrated that lipid metabolism is substantially reprogrammed in cancers [19]. Lipid uptake and storage are also elevated in malignant tumors [20]. These mechanisms further affect tumor biology, such as immune escape and cellular invasion [21]. Cholesterol and triglycerides are insoluble in water and therefore these lipids must be transported in association with lipoproteins. Plasma lipoproteins can be divided into seven classes, including *chylomicrons*, *chylomicron remnants*, *VLDL*, *IDL*, *LDL*, *HDL*, and *Lp (a)*. *VLDL* is responsible for carrying *triacylglycerol* synthesized in the liver to peripheral tissues for utilization. The surface proteins of *VLDL* include apolipoprotein (Apo) B-100, Apo C-I, Apo C-II, Apo C-III, and Apo E. Among these, Apo B-100 is the core structural protein and is produced by the liver [22]. A research revealed that *VLDL* levels were elevated in lung cancer patients compared to non-cancer subjects [23]. In addition, *VLDL* promote breast cancer progression and metastasis through Akt-induced (epithelial–mesenchymal transition) EMT and angiogenesis [24]. To the best of our knowledge, the effect of *VLDL* on OC has not been studied. And we found that *medium VLDL* may promotes OC oncogenesis. This could be due to higher concentrations of *VLDLs* in the blood are often translated into higher levels of atherogenic particles and *LDLs*. *VLDL* uptake brings lipids and offers a sustainable source of energy for cancer cells [25].

*Androsterone sulfate* was the most abundant 5 alpha-reduced androgen metabolite in serum. *Androsterone sulfate* was identified as independent variables associated with the incidence of hepatocellular carcinoma (HCC) and non-alcoholic fatty liver disease (NAFLD) advanced fibrosis [26, 27]. *Androsterone sulfate* seem robust to predict the abortion rate of the polycystic ovary syndrome (PCOS) group, with an AUC of 0.941 [28]. Our research showed *androsterone sulfate* may be the risk factor for OC. The underlying mechanism leading to the adverse outcome remain unclear.

*Acylcarnitines* are esters arising from the conjugation of fatty acids with *L-carnitine*. They are widely used and produced in cellular energy metabolism pathways. The well established biologic function of *acylcarnitine* is to transport acyl groups from the cytosol into the mitochondrial matrix for β-oxidation, leading to the production of energy to sustain cell activity [29]. *Acylcarnitine* had being identified as important indicators in metabolic studies of many diseases, including metabolic disorders, cardiovascular diseases, diabetes, depression, neurologic disorders, and certain cancers [30]. In a previous study, *propionylcarnitine* was significantly associated with lung cancer incidence [31]. Chen J et al. suggested that elevated *propionylcarnitine* was considered as potential biomarker candidates to diagnosed EOC [10]. Our results were consistent with the above.

In contrast to *propionylcarnitine*, *octanoylcarnitine* was gradually diminished in patients with HCC, and combining AFP with the metabolic biomarkers included *octanoylcarnitine* slightly increased the AUC value in the test set to 0.97 [32]. Our results also showed that *octanoylcarnitine* was negatively associated with OC. To date, the expression of *octanoylcarnitine* in different tumors remain conclusive [33], the expression in OC needs to be further verified. We also found that another acylcarnitine metabolite (*cis-4-decenyl carnitine*) was negatively correlated with OC. Studies of *cis-4-decenyl carnitine* in tumors are still lacking.

In addition to *medium VLDL* and *acylcarnitine*, we also found another two potential metabolites biomarker. The study of *5alpha-androstan-3beta-17beta-diol disulfate* was limited to the field of Alzheimer’s disease (AD) [34]. And elevated serum *N2, N2-dimethylguanosine* was used for chronic kidney disease (CKD) diagnosis [35]. Urinary excretion of *N2,N2-dimethylguanosine* by adults was used for acute leukemia follow-up [36].

There are still some limitations in this study. Firstly, this study utilized GWAS data of the same ethnicity (European population) as both the exposure and outcome groups in the Mendelian randomization analysis, aiming to minimize potential confounding effects of ethnicity on the outcome. However, this approach introduces limitations to the generalizability of the findings, as the conclusions may be subject to bias when extrapolated to the entire human population due to racial factors. Secondly, this study has yet to provide evidence regarding the mechanisms and molecular pathways through which metabolites influence the development of OC. Ultimately, the statistical power associated with the metabolites in this study is generally low, suggesting an increased likelihood of incurring Type II errors. Upon meticulous review, we infer that this is attributed to the disproportionately low number of cases in the outcome (ovarian cancer). The reason for the low proportion of cases in the dataset should be attributed to the relatively low incidence rate of ovarian cancer. These limitations will guide our future research directions and drive continuous improvement.

## Conclusion

To summarize, our study revealed the complex effect of metabolites on OC through Mendelian randomization. As promising biomarkers, these metabolites are worthy of further clinical validation.

### Electronic supplementary material

Below is the link to the electronic supplementary material.

**Supplementary Material 1**. Detailed Information of 637 Human Metabolite Data

**Supplementary Material 2**. List of Filtered SNPs

**Supplementary Material 3**. FDR-adjusted P-values of 31 Metabolites

**Supplementary Material 4**. IVW Analysis Results and Statistical Power Values for 31 Metabolites

**Supplementary Material 5**. Results of PhenoScanner

## Data Availability

The data that support the findings of this study are available from the corresponding author upon reasonable request.

## Abbreviations

AD: Alzheimer’s disease
CA125: Serum cancer antigen 125
CKD: Chronic kidney disease
EOC: Epithelial ovarian cancer
EMT: Epithelial-mesenchymal transition
GWAS: Genome-wide association study
HCC: Hepatocellular carcinoma
IVs: Instrumental variables
IVW: Inverse variance-weighted method
MR: Mendelian randomization
NAFLD: Non-alcoholic fatty liver disease
OC: Ovarian cancer
PCOS: Polycystic ovary syndrome
RCT: Randomized controlled trial
SNP: Single nucleotide polymorphisms
TSMR: Two-Sample Mendelian Randomization
VLDL: Very-low-density lipoprotein
WM: Weighted median

## References

[1] Ali AT, Al-Ani O, Al-Ani F (2023). Epidemiology and risk factors for ovarian cancer. Przeglad Menopauzalny = Menopause Review.

[2] Aus AT (2020). Can we prevent ovarian cancer?. Ceska Gynekol.

[3] Siegel RL, Miller KD, Fuchs HE, Jemal A. Cancer statistics, 2021. Cancer J Clin. 2021;71(1):7–33. 10.3322/caac.21654 33433946

[4] Liu J, Matulonis UA (2014). New strategies in ovarian cancer: translating the molecular complexity of ovarian cancer into treatment advances. Clin cancer Research: Official J Am Association Cancer Res.

[5] Petricoin EF, Ardekani AM, Hitt BA, Levine PJ, Fusaro VA, Steinberg SM (2002). Use of proteomic patterns in serum to identify ovarian cancer. Lancet.

[6] Hanahan D, Weinberg RA (2011). Hallmarks of cancer: the next generation. Cell.

[7] Kipps E, Tan DS, Kaye SB (2013). Meeting the challenge of ascites in ovarian cancer: new avenues for therapy and research. Nat Rev Cancer.

[8] Jacob M, Lopata AL, Dasouki M, Abdel Rahman AM (2019). Metabolomics toward personalized medicine. Mass Spectrom Rev.

[9] Fan L, Yin M, Ke C, Ge T, Zhang G, Zhang W (2016). Use of plasma metabolomics to identify diagnostic biomarkers for early stage epithelial ovarian Cancer. J Cancer.

[10] Chen J, Zhang X, Cao R, Lu X, Zhao S, Fekete A (2011). Serum 27-nor-5β-cholestane-3,7,12,24,25 pentol glucuronide discovered by metabolomics as potential diagnostic biomarker for epithelium ovarian cancer. J Proteome Res.

[11] Zeleznik OA, Clish CB, Kraft P, Avila-Pacheco J, Eliassen AH, Tworoger SS (2020). Circulating lysophosphatidylcholines, Phosphatidylcholines, ceramides, and Sphingomyelins and Ovarian Cancer risk: a 23-Year prospective study. J Natl Cancer Inst.

[12] Burgess S, Scott RA, Timpson NJ, Davey Smith G, Thompson SG (2015). Using published data in mendelian randomization: a blueprint for efficient identification of causal risk factors. Eur J Epidemiol.

[13] Burgess S, Swanson SA, Labrecque JA (2021). Are mendelian randomization investigations immune from bias due to reverse causation?. Eur J Epidemiol.

[14] Guo JZ, Xiao Q, Gao S, Li XQ, Wu QJ, Gong TT (2021). Review of mendelian randomization studies on ovarian Cancer. Front Oncol.

[15] Brion MJ, Shakhbazov K, Visscher PM (2013). Calculating statistical power in mendelian randomization studies. Int J Epidemiol.

[16] Kamat MA, Blackshaw JA, Young R, Surendran P, Burgess S, Danesh J (2019). PhenoScanner V2: an expanded tool for searching human genotype-phenotype associations. Bioinformatics.

[17] Shin SY, Fauman EB, Petersen AK, Krumsiek J, Santos R, Huang J (2014). An atlas of genetic influences on human blood metabolites. Nat Genet.

[18] Kettunen J, Demirkan A, Würtz P, Draisma HH, Haller T, Rawal R (2016). Genome-wide study for circulating metabolites identifies 62 loci and reveals novel systemic effects of LPA. Nat Commun.

[19] Schulze A, Harris AL (2012). How cancer metabolism is tuned for proliferation and vulnerable to disruption. Nature.

[20] Geng F, Guo D. Lipid droplets, potential biomarker and metabolic target in glioblastoma. Internal medicine review (Washington, DC:, Online.). 2017;3(5). 10.18103/imr.v3i5.443 PMC5639724 29034362

[21] Long J, Zhang CJ, Zhu N, Du K, Yin YF, Tan X (2018). Lipid metabolism and carcinogenesis, cancer development. Am J cancer Res.

[22] Feingold KR. Introduction to lipids and lipoproteins. In: Feingold KR, Anawalt B, Blackman MR, Boyce A, Chrousos G, Corpas E, et al. editors. Endotext. South Dartmouth (MA): MDText.com, Inc.Copyright © 2000–2023. MDText.com, Inc.; 2000. 26247089

[23] Şahin F, Aslan AF. Relationship between inflammatory and biological markers and Lung Cancer. J Clin Med. 2018;7(7). 10.3390/jcm7070160 PMC6069225 29941786

[24] Lu CW, Lo YH, Chen CH, Lin CY, Tsai CH, Chen PJ (2017). VLDL and LDL, but not HDL, promote breast cancer cell proliferation, metastasis and angiogenesis. Cancer Lett.

[25] Lupien LE, Bloch K, Dehairs J, Traphagen NA, Feng WW, Davis WL (2020). Endocytosis of very low-density lipoproteins: an unexpected mechanism for lipid acquisition by breast cancer cells. J Lipid Res.

[26] Jee SH, Kim M, Kim M, Yoo HJ, Kim H, Jung KJ (2018). Metabolomics Profiles of Hepatocellular Carcinoma in a Korean prospective cohort: the Korean Cancer Prevention Study-II. Cancer prevention research (Philadelphia. Pa).

[27] Caussy C, Ajmera VH, Puri P, Hsu CL, Bassirian S, Mgdsyan M (2019). Serum metabolites detect the presence of advanced fibrosis in derivation and validation cohorts of patients with non-alcoholic fatty liver disease. Gut.

[28] Guan SY, Liu YY, Guo Y, Shen XX, Liu Y, Jin HX (2022). Potential biomarkers for clinical outcomes of IVF cycles in women with/without PCOS: searching with metabolomics. Front Endocrinol.

[29] Indiveri C, Iacobazzi V, Tonazzi A, Giangregorio N, Infantino V, Convertini P (2011). The mitochondrial carnitine/acylcarnitine carrier: function, structure and physiopathology. Mol Aspects Med.

[30] Dambrova M, Makrecka-Kuka M, Kuka J, Vilskersts R, Nordberg D, Attwood MM (2022). Acylcarnitines: nomenclature, biomarkers, therapeutic potential, drug targets, and clinical trials. Pharmacol Rev.

[31] Zhang X, Wang C, Li C, Zhao H (2023). Development and internal validation of nomograms based on plasma metabolites to predict non-small cell lung cancer risk in smoking and nonsmoking populations. Thorac cancer.

[32] Kim DJ, Cho EJ, Yu KS, Jang IJ, Yoon JH, Park T et al. Comprehensive Metabolomic search for biomarkers to Differentiate Early Stage Hepatocellular Carcinoma from Cirrhosis. Cancers. 2019;11(10). 10.3390/cancers11101497 PMC6826937 31590436

[33] Chen L, Wang S, Zhang Y, Li Y, Zhang X, Ma J (2022). Multi-omics reveals specific host metabolism-microbiome associations in intracerebral hemorrhage. Front Cell Infect Microbiol.

[34] Sun L, Guo D, Jia Y (2022). Association between Human Blood Metabolome and the risk of Alzheimer’s Disease. Ann Neurol.

[35] Su D, Chen J, Du S, Kim H, Yu B, Wong KE (2023). Metabolomic markers of Ultra-processed Food and Incident CKD. Clin J Am Soc Nephrology: CJASN.

[36] Heldman DA, Grever MR, Trewyn RW (1983). Differential excretion of modified nucleosides in adult acute leukemia. Blood.

